# Effects of Sample Preparation on Particle Size Distributions of Different Types of Silica in Suspensions

**DOI:** 10.3390/nano8070454

**Published:** 2018-06-21

**Authors:** Rodrigo R. Retamal Marín, Frank Babick, Gottlieb-Georg Lindner, Martin Wiemann, Michael Stintz

**Affiliations:** 1 Research Group Mechanical Process Engineering, Institute of Process Engineering and Environmental Technology, Technische Universität Dresden, Münchner Platz 3, D-01062 Dresden, Germany; frank.babick@tu-dresden.de (F.B.); michael.stintz@tu-dresden.de (M.S.); 2 Evonik Resource Efficiency GmbH, Brühler Straße 2, 50389 Wesseling, Germany; gottlieb-georg.lindner@evonik.com; 3 IBE R&D Institute for Lung Health gGmbH, Mendelstr 11, D-48149 Münster, Germany; martin.wiemann@ibe-ms.de

**Keywords:** nanomaterials (NMs), nanostructured, synthetic amorphous silica (SAS), ultrasonic dispersing (USD), energy density, sample preparation, in vitro testing

## Abstract

The granulometric characterization of synthetic amorphous silica (SAS) nanomaterials (NMs) still demands harmonized standard operation procedures. SAS is produced as either precipitated, fumed (pyrogenic), gel and colloidal SAS and these qualities differ, among others, with respect to their state of aggregation and aggregate strength. The reproducible production of suspensions from SAS, e.g., for biological testing purposes, demands a reasonable amount of dispersing energy. Using materials representative for each of the types of SAS, we employed ultrasonic dispersing (USD) at energy densities of 8–1440 J/mL and measured resulting particle sizes by dynamic light scattering and laser diffraction. In this energy range, USD had no significant impact on particle size distributions of colloidal and gel SAS, but clearly decreased the particle size of precipitated and fumed SAS. For high energy densities, we observed a considerable contamination of SAS suspensions with metal particles caused by abrasion of the sonotrode’s tip. To avoid this problem, the energy density was limited to 270 J/mL and remaining coarse particles were removed with size-selective filtration. The ultrasonic dispersion of SAS at medium levels of energy density is suggested as a reasonable compromise to produce SAS suspensions for toxicological in vitro testing.

## 1. Introduction

Modification of physico-chemical properties of nanomaterials (NMs) or nanostructured materials allows the control and variation of design, development and improvement of new products. Synthetic amorphous silica (SAS) comprise an important group of NMs, which are added to industrial as well as consumer products such as cosmetic or foods [1,2,3,4,5,6,7] within which they serve e.g., as stabilizers, thickeners, pigments, flow enhancing agents, or UV absorbers [3,8,9,10]. Based on some concern regarding possible health impacts and safety risks of NMs, legal authorities request the toxicological analysis of SAS NMs by means of in vivo and in vitro studies [11,12,13,14,15]. 

Generally, ultrasonic dispersing or separation has been used for sample preparation of nanomaterials for safety assessment [16]. These studies can support the optimization of nanosynthesis or nano-applications for the sake of a “Green Synthesis of Nanomaterials”. One example for such green nano-application of amorphous silica in entomology and parasitology as a nanopesticide has been considered safe for humans because of the specific mechanisms of action [17]. 

An important aspect of exposure and toxicological analyses is the characterization of NMs with respect to particle size. Most SAS occur in an aggregated state with particle sizes ranging from nanometer-sized primary particles to micrometer-sized aggregates or agglomerates [18,19,20]. However, the sample preparation for a specific in vitro test should consider the particle size-distribution which is of relevance for a given exposure pathway [21,22,23,24]. For example, inhalation of particles into the human respiratory tract leads to a fractionation of particles: larger agglomerates are deposited in the nasopharyngeal region (5–30 μm), small agglomerates are partially deposited in the tracheobronchial region (1–5 μm), and only small (<1 μm) and nano-sized materials (1–100 nm) may penetrate into the alveolar region of the lung [25,26,27,28,29,30]. Thus, toxicity testing of NMs using in vitro lung models demands the preparation of properly suspended samples under defined conditions. 

In general, studies on environmental and health risk assessment focus on transport and deposition of NMs in real-life exposure scenarios. Both processes are governed by the mobility of aggregates and agglomerates, for which reason the size of aggregates and agglomerates needs to be measured. This is different from the nanomaterial definition recommendation of the European Commission, which is based on number-weighted distribution of the minimum size of isolated particles or constituent particles within aggregates and agglomerates [31,32].

The analysis of nanomaterials (NMs) and nanostructured materials requires standard operation procedures (SOPs) for the preparation of suspension samples to ensure defined granulometric states [33,34,35]. Therefore, the preparation and analysis of nanomaterial suspensions needs a high degree of standardization with respect to primary sample preparation (stock suspension), secondary sample preparation, conditioning (e.g., adjusting suspension composition or concentration), sample splitting and finally measurement/interpretation. All these steps need to be considered for the characterization of liquid-suspended powders and for the comparison of different SOPs in view of their reproducibility. Although wetting and low energy dispersion of SAS powders in the suspension are substantial components of (primary) sample preparation, further ultrasonic dispersing (USD) is needed as it is the most versatile method to disintegrate large particle agglomerates into small particle aggregates or primary particles. At the same time, stabilization and homogenization of the dispersed particles are necessary.

USD is a rather intense type of dispersing, which relies on the hydrodynamic stress caused by collapsing cavitation bubbles. As USD can be performed with different types of equipment (e.g., ultrasonic bath, high-power probe sonicator, or cup-horn sonication) methods ensuring its reproducibility are mandatory. Several studies have shown that the energy density (measured in J/mL) serves as a well-suited parameter for obtaining a largely identical degree of USD of nanomaterials among different laboratories [23,36,37]. Of note, the application of different ultrasonic dispersing methods (e.g., variation of the sonotrode geometry) and parameter settings (e.g., sonication time and vibration amplitude) requires the application of the energy density concept, applicable to ultrasonication, rotor-stator systems [37,38] or high-pressure dispersing [39,40,41]. In all these cases, the ultrasonic dispersing energy density can be used as a main parameter for comparing available and new sample preparation protocols or SOPs. 

To achieve a reproducible particle size analysis, it is necessary to use adequate sample preparation techniques. The USD facilitates the disintegration of submicron agglomerates, which are therefore of special relevance for the preparation of stable and homogeneous distribution of particles in the suspension and contribute to achieve a stable dispersion. The particles are under interaction of different dispersion forces which control their random dispersion in the sample volume. The energy density has been used in Table 1 to compare several studies, which are developed for the application to nanostructured materials).

Table 1 shows that the energy densities used in several recent studies to disperse nanomaterials differ by more than two orders of magnitude. This raises the question, as to which extent they influence the results of particle size measurement. Previous studies on different grades of NMs such as SiO_2_, Al_2_O_3_, or TiO_2_ have shown that even with a comparatively high energy density of up to 5 kJ/mL a maximum dispersion cannot be achieved for all materials [20]. However, as administration of such energy densities requires extensive cooling of the samples and prolonged periods of ultrasonic treatment, we were seeking for a reasonable compromise to achieve an acceptable dispersion of nanomaterials. 

In this paper, we examine the effect of USD energy on the dispersion of SAS and characterize resulting particle size distributions (PSD). Despite their identical chemical composition, SAS products show considerable variations with respect to the synthetic routes, particle morphology, and product properties. The synthesis of silica is realized either in aqueous solution based on sodium silicate solution or in gaseous phase from SiCl_4_ [48,49]. The types of silica originating from silica synthesis processes in aqueous solution are silica gel (SG), precipitated silica (PS) and colloidal silica (CS). Fumed silica (FS), also referred to as pyrogenic silica, is synthesized from gaseous phase. SAS products are nanostructured NMs [46] as they consist of aggregates and agglomerates of nanosized constituent particles (FS, PS and SG, cf. [50]) or well-dispersed nano-objects (CS). Accordingly, the preparation of suspensions of FS, PS and SG requires defined dispersion procedures for their use, e.g., in toxicity studies, and characterization, whereas this is not really necessary for colloidal silica [19,20]. Furthermore, the different types of silica have a characteristic morphology due to their varied synthetic processes. This is an important issue that needs to be considered for comparison and data interpretation (e.g., reproducibility, effectiveness).

## 2. Materials and Methods

### 2.1. Materials

This study analyzed SAS products, representative for FS, PS, SG and CS, respectively. While FS, PS, SG were provided as untreated hydrophilic powders, CS was provided as an aqueous suspension. Important physico-chemical properties are summarized in Table 2.

**Figure 1:**
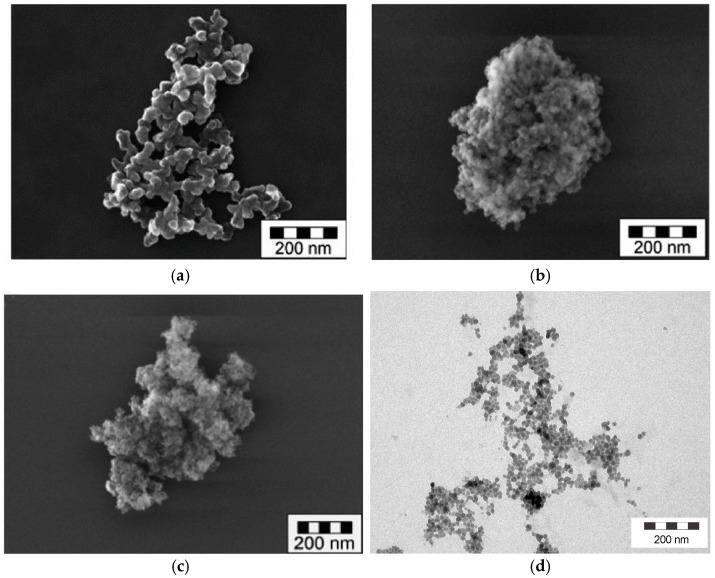
SEM and TEM images of different silica types. (**a**) fumed (pyrogenic) silica (opened fractal-like aggregates), (**b**) precipitated silica (compact fractal-like aggregates), (**c**) silica gel (compact and microporous fractal-like aggregates) and (**d**) colloidal silica (isolated spherical nanoparticles or small aggregates, here dried on TEM grid to opened agglomerates).

Figure 1a–c show SEM (JEOL Ltd, Tokyo, Japan) images of typical aggregates of FS, PS and SG [50]. In contrast, CS (Figure 1d) contains isolated spherical nanoparticles, which have gathered into an agglomerate-like structure upon drying on the TEM grid.

### 2.2. Instruments and Procedures for Sample Preparation

To prepare stock suspensions of SAS 1 wt.-% of silica powders (FS, PS, SG) were dispersed in 100 mL de-ionized water (18.3 MΩcm, 0.2 μm filtered). To avoid re-agglomeration, the particles were placed in a liquid environment that ensured high surface charges. The pH value of prepared silica suspension is far from the isoelectric point of silica (e.g., pH 1.8–2.5) and it has a low electric conductivity (see Table 2) [53,54,55]. Powders were dispersed by different treatments: Firstly, by means of a paddle stirrer (PS) (model RW 11 basic, IKA, Staufen, Germany), which administered the lowest input of mechanical energy into the suspension and which was used for the homogenization of SAS suspensions. Of note, the geometric size of the paddle stirrer and the sample beaker as well as stirring velocity ensured hydrodynamic equivalence to the “paddle apparatus” specified in Ph. Eur. 5.7. (2006) [56]. Secondly, by means of a turbulent shear rotor stator (RS) (Ultra-Turrax T25, IKA) which was used to achieve advanced dispersion and to investigate changes of PSD of different SAS types upon progressive dispersion energy. The RS provides shear forces, which cause shear stress on particle agglomerates. Thirdly, by means of immersion horns (three different instruments, see Table 3) which were used in most experiments [33,34,35]. The sonotrode or horn is in direct contact with the suspension and the dispersion effect is associated to cavitation, which occurs in highly intensive sound fields. Furthermore, the cavitation causes the formation of vapor cavities in a liquid (bubbles) which steadily grow to a critical size, at which they turn instable and implode [20]. This implosion produces high temperature and rapid micro-jets, which exert mechanical stress to the particles close to the formed bubble [57]. This mechanical stress leads to the fragmentation or at the least to the erosion upon the direct contact with the imploding cavities [58,59].

The USD equipment consisted of three different instruments equipped with different sonotrodes (a few mm up to a few cm) (see Table 3), which were operated at frequencies in the range of 20 kHz to 100 kHz. Instruments had a nominal power consumption of a few Watt to approximately 1 kW [18]. The calorimetric energy input was measured at different dispersing instruments. Most of them work at frequencies in the range of 20 kHz. In pilot experiments, various tip diameters of the sonotrodes were selected according to the geometry of the beaker and the sample volume required by the sample preparation protocols.

For the particle size analysis of all silica types in this study one USD equipment was selected, the generator Vibra-Cell 72412 (Sonics and Materials; 20 kHz, nominal power: 600 W together with a 19 mm tip diameter solid probe. The tip of the probe was replaced for each series of dispersing experiments. USD was performed at maximum amplitude (100%) in a pulsed mode (2s:2s) with the probe being uniformly immersed in the sample. The same type of glass beaker was employed for all samples; the beakers were placed in cooled water during the USD. Even though, samples were steadily heated-up with ongoing USD, for which reason the USD was interrupted after a maximum of 4 min to cool down the complete the sample and the ultrasonic probe. This procedure ensured that the sample temperature stayed below 33 °C [20].

### 2.3. Instruments for Particle Size Analysis

USD leads to deagglomeration and disintegration of aggregates and the corresponding change in the granulometric state was quantified by laser diffraction (LD) and dynamic light scattering (DLS). However, these standard analytical techniques are based on mathematical models, which are not perfectly applicable to the examined particle systems, e.g., because they assume spherical particles (e.g., Stokes-Einstein relation and Mie theory for DLS), or because they do not cover the whole size range of broad distributions (e.g., Fraunhofer diffraction theory for LD) [18,19,20,60,61]. To avoid these technical limitations and for a better interpretation of measured data it is advantageous to apply both techniques to well-adapted dilutions of the same sample.

LD measurements were carried out with a HELOS KR (Sympatec, Clausthal-Zellerfeld, Germany) for angular ranges below 35° (i.e., forward scattering). Within this study an angular range of 0.1° to 9° (measurement range R3) was used, which is sensitive for particles of 0.5 μm to 175 μm, but which is insensitive to nanoparticles (x ≤ 100 nm). These measurements were rather insensitive to small, weakly scattering particles << 1 μm. 

To quantify particle sizes in sub-micrometer range, DLS measurements were conducted. The employed instrumentation, HPPS (Malvern, UK), bases on backscattered (173°) and sideward scattered light, respectively. Measured DLS signals (i.e., autocorrelation functions) were analyzed with inversion procedures, which compute complete size distributions, and cumulant analysis. The latter yields a polydispersity index, PDI, and a characteristic mean particle size, xcum, which is the harmonic mean of the intensity-weighted size distribution. The samples are filled in closed cuvettes (4 mL), which are placed in the temperature-controlled sample holder at least 15 min before the measurements.

### 2.4. Estimation of the Calorimetric Energy Input

The effectivity of the ultrasonication, in comparison with other dispersion procedures regarding size reduction, demands the necessity to evaluate the applied acoustic energy per unit suspension volume (*E_V_*) [18,20,23,37,46]. The calculation of ultrasonic dispersion energy cannot be calculated directly. There are two ways to estimate the inserted acoustic energy. One way is the estimation from electrical energy consumption (*E_V_*_,el_) and the other possibility is through the generated heat after implosion of bubbles (*E_V_*_,cal_). The electrical energy consumption depends on many factors: transformation of energy of ultrasonic dispersions instruments (e.g., normal capacity, range of frequencies, types and probe diameter) that must be considered for comparison and validation. Furthermore, another important point to consider is the acoustic reflections inside the probe depending on, for example, sample volume, density and the propagation velocity of sound of liquid (acoustic impedance) [62]. 

This study uses the calorimetric energy input as decisive parameter for the effect of USD. Therefore, the USD devices needed to be calibrated regarding the calorimetric power input for the setup and settings (sample volume, horn diameter; nominal ultrasonic amplitude) employed in dispersing the SAS samples. The calibration comprises the evaluation of temperature increase by ultrasonication of a defined volume of de-ionized water. In this study, USD was conducted at 100 mL suspension sample placed in a 150 mL cylindrical borosilicate beaker. Please note that other studies worked with higher volumes (e.g., 500 mL, [23,34]) or smaller ones (e.g., 6 mL [33,42,43]). A further difference to other studies is that the beaker was placed in insulating foam, to minimize heat exchange with environment. The whole setup, including the ultrasonic probe as well as a thermometer with short response-time, was allowed to thermally equilibrate. Then ultrasonication was started and the temperature within the beaker was recorded as function of time. 

The calorimetric energy input into a suspension sample by ultrasonication can be calculated from the heat production rate *P*_cal_ which is a function of the dispersion time *t*_disp_. The former parameter is valid for defined conditions of ultrasonication, i.e., for defined sample properties and USD settings. It can be determined by specifically designed experiments that measure the temperature increase when sonicating the particle-free dispersion medium (e.g., water). Furthermore, it is necessary to consider the mass and specific heat of the beaker to have a correct determination of heat production and to evaluate the initial slope of temperature increase, which needs to be less than 4 Kelvin (Δ*T* ≤ 4 K). Furthermore, the following proposed equation to estimate the calorimetric energy input assumes that the sonotrode has a zero heat capacity and that there is no heat exchange with the environment:

(1) $$P_{\mathrm{cal}}=(m_{w}c_{p,w}+m_{b}c_{p,b})\cdot \Delta T/t_{\mathrm{disp}}$$

where *m_w_* denotes the mass of liquid, *m_b_* denotes the mass of beaker, *c*_p,w_ specific heat of water, *c*_p,b_ specific heat of beaker, ∆*T* the temperature increase and *t* the dispersion time [63,64]. The calorimetric energy density can be determined as follows:

(2) $$E_{V,\mathrm{cal}}=\frac{P_{\mathrm{cal}}\cdot t_{\mathrm{disp}}}{V}=(m_{W}c_{p,W}+m_{B}c_{p,B})\cdot \Delta T/V$$

with *V* being the suspension volume.

The energy density is considered to be a most important process parameter when dispersing suspensions and emulsions. Frequently, a power-law relationship can be established between average particle size *x* and energy density *E_V_*_,cal_ (e.g., [47]):

(3) $$\bar{x}\propto E_{V,\mathrm{cal}}^{-b}$$

where “average particle size” can be any characteristic distribution parameter (e.g., median size or arithmetic mean) referring to a defined type of quantity, in which the size distribution is weighted (e.g., number, volume or scattering intensity). The exponent b describes the material’s dispersibility under the specified conditions. 

Finally, there are some assumptions to estimate values for calorimetric calibration of ultrasonic instruments. The first assumption is the uniform temperature of water and beaker, second—zero heat capacity of ultrasonic probe and third—no heat transfers out of the system beaker-water.

## 3. Results and Discussion

### 3.1. Calorimetric Calibration of Probe Sonication

**Figure 2:**
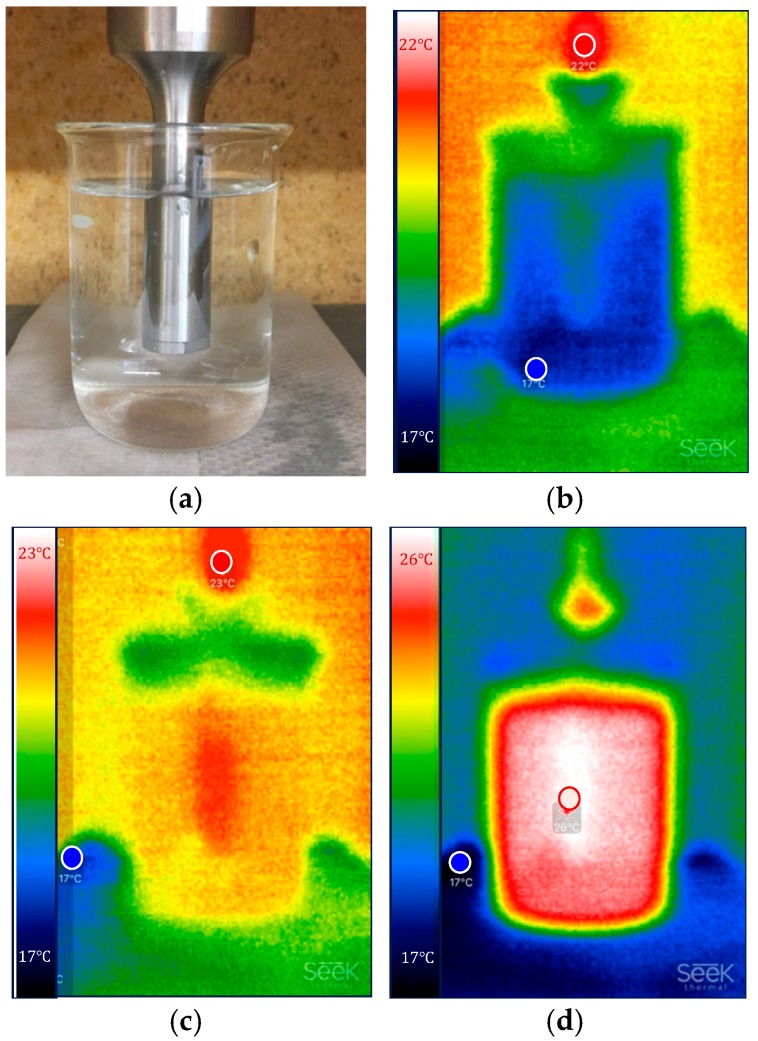
Progressive heating of the sonotrode, sample fluid, and beaker in the course of USD. Images were captured with a thermal imaging camera (Seek Thermal) after different dispersing periods; (**a**,**b**): 0 s, (**c**): 30 s, (**d**): 60 s. Temperature (in degree Celsius) is shown on a pseudo-color scale whose range was automatically adjusted to the temperature peak (T_min_/T_max_) of the measurement (i.e., (**a**) 17 °C/22 °C, (**b**) 17 °C/23 °C, (**c**) 17 °C/26 °C).

While the ultrasonic wave propagates through the dispersion medium, its energy is absorbed and converted into heat [65,66]. Figure 2 exemplifies this progressive heating during USD for a non-insulated beaker. The images give an impression of how the heat generated upon ultrasonication is transferred from the sonotrode into the surrounding medium and associated masses. Therefore, it is important to consider some issues such as sample volume, or isolating foam to achieve a correct calorimetric calibration of probe sonication, as explained in the proposed protocol (see Section 2.4). 

**Figure 3:**
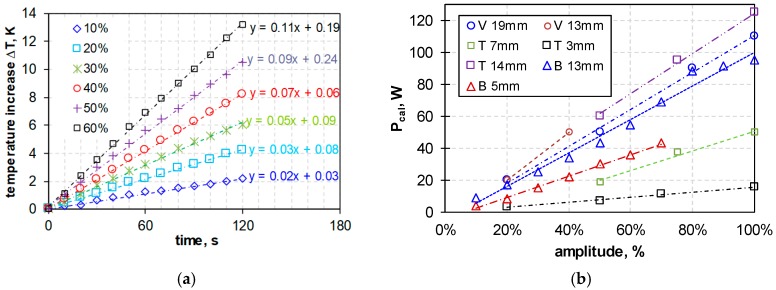
Calorimetric calibration curves of ultrasonic dispersing instruments. (**a**) temperature increases (Δ*T*) in Kelvin (K) over time, obtained with a 13 mm tip diameter sonotrode (Branson 450D) and increasing amplitudes (10–60% from maximum, as indicated in the graph). (**b**) Heat production (*P*_cal_, W) of the different dispersing instruments and sonotrode geometries Vibra-Cell 72412 (V), Topas UDS751 (T) and Branson SONIFIER 450F (B) (outlined in Table 3) as a function of increasing amplitude (in % from maximum).

The calorimetric calibration was calculated for different ultrasonic dispersing instruments (according to the protocol in Section 2.4) to compare their heat production after setting of different parameters (see Section 2.2). The calorimetric measurements delivered the results in Figure 3.

### 3.2. Sample Preparation by Probe Sonication

#### 3.2.1. Impact of USD on Particle Size Distribution of SAS

Previous studies have shown that the PSD of, e.g., FS may be highly polydisperse [20,54]) and covers a wide range from of a few nanometers up to several micrometers [18,19,20]. As outlined above (Section 2.3), the appropriate granulometric analysis of such samples requires a combination of LD and DLS. This section addresses the effectiveness of ultrasonic dispersing on particle size of silica types measured with both techniques.

Suspensions of the SAS types were prepared by a combination of dispersing procedures and defined calorimetric energy densities (EV, J/mL). Depending on the instrumentation (see Section 2.3) dispersing energies were weak from propeller stirrer (PS), moderate from rotor-stator (RS), or intense from ultrasonication (US, different dispersing energies) and the selected calorimetric energy densities’ values are based on the calorimetric calibration of probe sonication (see Figure 3). The calorimetric energy density of the US treatment ranged from 8 J/mL through 18 J/mL and 270 J/mL to a maximum energy input of 1440 J/mL. This stepwise increase of energy input allows for a comprehensive characterization of SAS with respect to particle size and morphology. 

**Figure 4:**
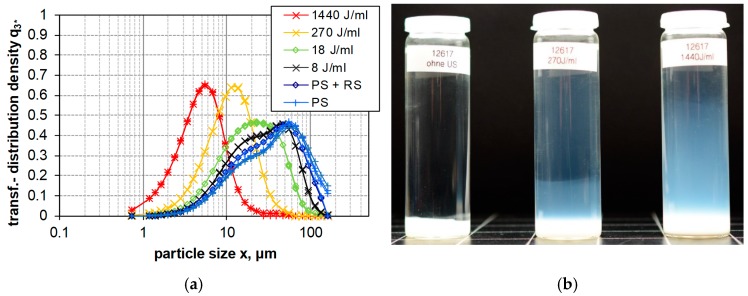
Particle size distribution of PS (440 m^2^/g) dispersed by different procedures. (**a**) Size distribution measured by laser diffraction spectroscopy plotted as the transformed distribution density (q^3^*). Weak (PS), moderate (RS) and intense (US) dispersion with increasing ultrasonic energies (indicated in the diagram) were employed. (**b**) Silica suspensions after five months (left 1 J/mL, middle 270 J/mL, and right 1440 J/mL).

**Figure 5:**
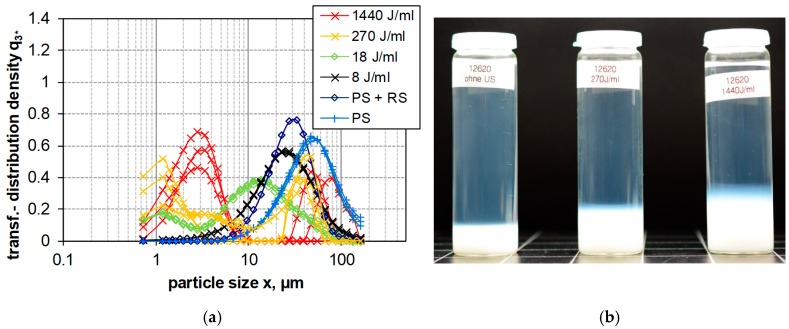
Particle size distribution of FS (300 m^2^/g) dispersed by different procedures. (**a**) Size distribution measured by laser diffraction spectroscopy plotted as the transformed distribution density (q^3^*). Weak (PS), moderate (RS) and intense (US) dispersion with increasing ultrasonic energies (indicated in the diagram) were employed. (**b**) Silica suspensions after five months (left 1 J/mL, middle 270 J/mL, and right 1440 J/mL).

**Figure 6:**
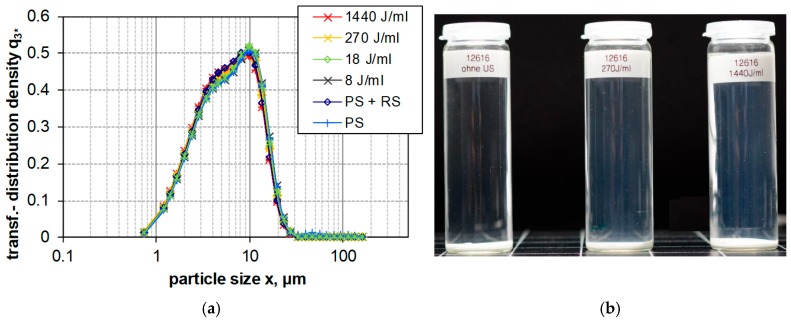
Particle size distribution of SG (BET: 700 m^2^/g) dispersed by different procedures. (**a**) Size distribution measured by laser diffraction spectroscopy plotted as the transformed distribution density (q^3^*). Weak (PS), moderate (RS) and intense (US) dispersion with increasing ultrasonic energies (indicated in the diagram) were employed. (**b**) Silica suspensions after five months (left 1 J/mL, middle 270 J/mL, and right 1440 J/mL).

Figure 4, Figure 5 and Figure 6 show the LD measurements of the PSD results of the transformed distribution density of upon increasing *E_V_*_,cal_ for PS, FS, and SG (see Section 2.1). The transformed distribution density represents in accordance with ISO 9276-1:1998 provide the differential size distribution on a log scaled abscissa. Areas under the curve represent the volume portions of the size classes [67]. The result of a long-term sedimentation of silica suspensions (after five months) is shown in parallel.

Precipitated silica (PS, 400 m^2^/g, 1 wt.-%) shows a clear tendency of deagglomeration by increasing dispersion energy (Figure 4a). Especially the presence of coarse, micrometer-sized agglomerates was diminished, and this effect started mostly at 18 J/mL. In line with these results the sedimentation profiles of ultrasonicated PS suspensions show an increasing degree of opacity upon 270 J/mL and 1440 J/mL (Figure 4b), suggesting the presence of slowly settling, light scattering particles in the sub-micrometer range. However, although the zone of opacity was wider upon 1440 J/mL, the volume of the white matter at the bottom was similar.

Largely similar to PS was fumed (pyrogenic) silica (FS, 300 m^2^/g, 1 wt.-%) deagglomerated by increasing USD energy (Figure 5a). The deagglomeration of large particles was achieved already with a moderate dispersion (RS) and increased further upon administration of USD energy. Interestingly, the mono-modal PSD of FS became bimodal upon 18, 270, and 1440 J/mL, such that a fine and a large fraction could be distinguished (Figure 5a). Of note, the large fraction generated by 270 J/mL and 1440 J/mL comprised larger particles as compared to the fraction induced by 18 J/mL. This suggests that elevated energy levels at least in part can provoke an agglomeration of FS. The effect was most obvious at an USD energy of 270 J/mL. These results confirm previous studies, where the coarser particles appeared at energy density levels above 171 J/mL [20]. The sedimentation profiles of FS at higher dispersion energies showed a similar degree of opacity in the supernatant, but an increased volume of the white sediment. This suggests that high USD energy leads to the formation of larger particles from the same mass of FS (see Figure 5b).

As can be seen in Figure 6a, the PSD of silica gel (SG, 700 m^2^/g, 1 wt.-%) remained unchanged upon increasing energy density. SG consists of compact (dense) and microporous fractal-like aggregates (see Figure 1), which appear to be insensitive to high USD energies and undergo a rapid sedimentation of particles (by median x_50,3_ = 6 μm), irrespective of USD treatment (see Figure 6b), although both ultrasonic treatments led to a similar degree of opacity of the supernatant which may be indicative of smaller particles (not measurable by LD).

**Figure 7:**
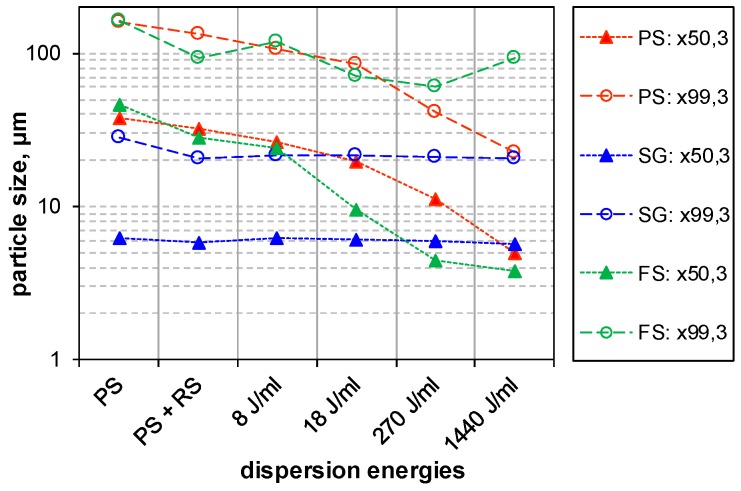
Particle size distribution of silica suspension after administration of increasing dispersion energy as measured by laser diffraction. Curves show the trends for the x_50,3_ and x_99,3_ quantiles of the volume weighted size distribution. USD energy density of ultrasonic treatment is indicated in J/mL.

**Figure 8:**
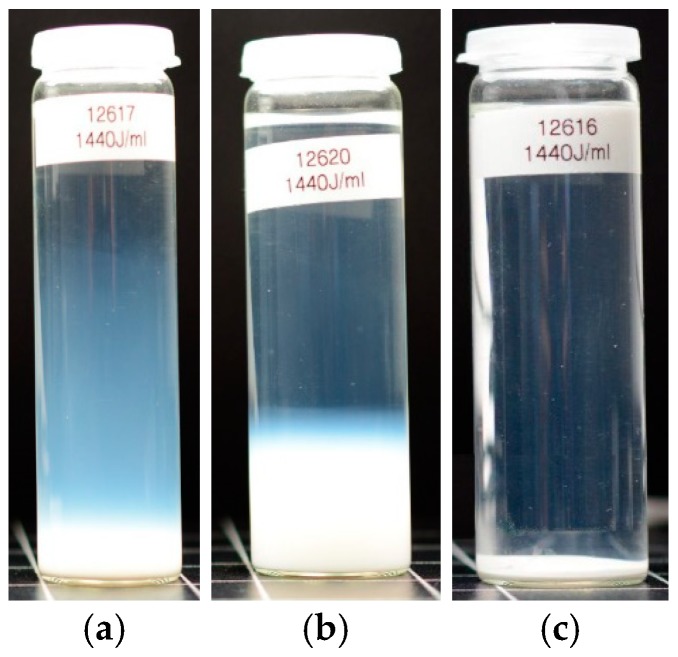
Intensely dispersed SAS samples (USD, *E_V_*_,cal_: 1440 J/mL) after long-term sedimentation (5 months): (**a**) PS; (**b**) FS; (**c**) SG.

The effect of increasing dispersion energy on particle size of PS, SG and FS, as measured by LD, is compared in Figure 7, which shows the trend analysis for the x_50,3_ and x_99,3_ quantiles of the volume weighted size distribution. Whereas the particle size of SG is not altered by increased dispersion energy, particle size of PS is constantly lowered. In the case of FS, only the x_50,3_ value reflects the decrease in particle size, whereas the x_99,3_ is inconsistent and shows an upwards trend demonstrating the coarsening or reagglomeration upon high USD energy. Figure 8 shows a comparison the stability of intensely dispersed silica types at 1440 J/mL after long-term sedimentation (5 months). The different sediment degrees of silica types support the LD results and provide a subjective information about sedimentation velocity in the gravitational field of the earth.

**Figure 9:**
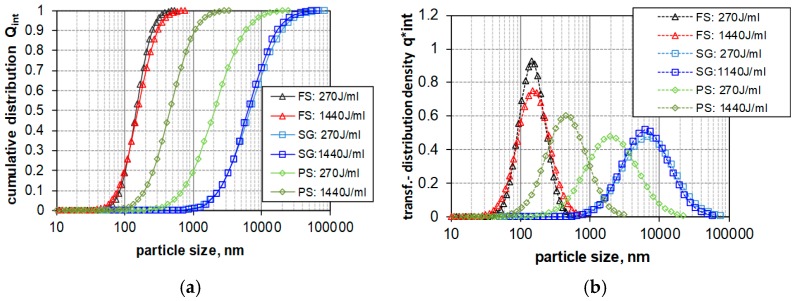
Particle size distribution (PSD) of amorphous silica suspensions dispersed by two different ultrasonic dispersion energy densities (270 and 1440 J/mL). PSD was measured by dynamic light scattering (DLS). FS: fumed silica, SG: gel silica, PS: precipitated silica. (**a**) Intensity-weighted sum functions for different energy density values. (**b**) Intensity-weighted transformed distribution density functions for different energy density values. Logarithmic normal distribution.

Since LD is not sensitive for silica particles smaller than 1 μm [68], the characterization of sub-micrometer particles (1 nm–10 μm) was carried out with DLS. Data of three silica types (FS, SG, PS) was expressed as intensity-weighted size distribution, using the characteristic values mean size (xcum) and the polydispersity index (PDI) obtained by cumulant analysis. Figure 9a compares the as result calculated logarithmic normal distribution (LND) and shows the impact of 270 J/mL and 1440 J/mL on particle size. In Figure 9b the Intensity-weighted transformed distribution density functions are shown. While the particle size of SG and FS remains nearly unchanged in the lower size range, the long-term sedimentation e cumulative size distribution curve of PS is shifted leftwards, indicating that particle size had shifted to submicron and nano range (<1 μm). Fumed silica (FS) shows a minimal tendency to increase the size of submicron particles upon high energy density.

**Figure 10:**
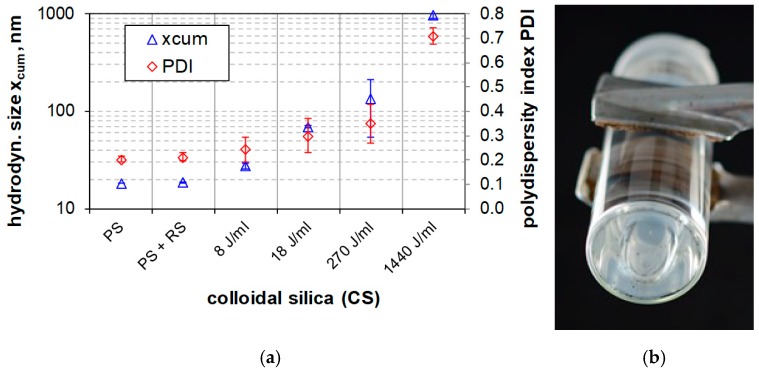
Particle size distribution of colloidal silica dispersed by different procedures measured by DLS. (**a**) Mean particle size (xcum) and corresponding polydispersity index (PDI) as determined with cumulant analysis. (**b**) Bottom view of a vial with an intensely dispersed colloidal silica sample (*E_V_*_,cal_: 1440 J/mL) taken after 5 months of gravitational settling.

Figure 10a shows the granulometric state of colloidal silica (CS) over the full range of dispersion energies as used in Figure 7. While stirring (PS, PS + RS) had no effect on particle size, ultrasonic treatment surprisingly led to a larger and broader PSD indicated by an increase in hydrodynamic size and PDI. The effect started at a low energy density of 8 J/mL and was found to be strongly augmented upon higher USD energies. We found that wear particles from the sonotrode’s tip, the larger of which appeared as a sediment at the bottom of the vial (Figure 10b), made a major contribution to this effect. Due to the strong light scattering properties of such metal particles (compared to the small and weakly scattering CS particles), even low amounts of wear particles contaminate the light optic measurements. If this increase of the mean particles size would be caused by agglomerated colloidal silica particles, they would be visible as a sediment layer after 5 months. In the case of silica gel scattering, intensity of the micrometer particles hides the contamination signals during measurement whereas, after settling, the contamination is embedded in the silica sediment.

#### 3.2.2. Sample Contamination with Probe Sonication

**Figure 11:**
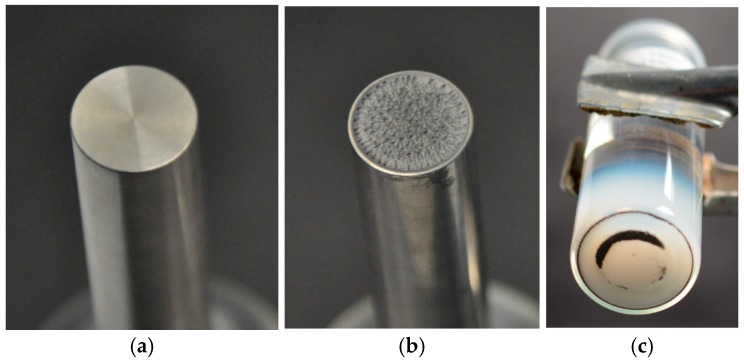
Comparison of new and used sonotrode with sonotrode abrasion as a consequence of long ultrasonic dispersion time (**a**,**b**); sonotrode abrasion sediment on the bottom of a precipitated silica suspension sample after high USD energy (**c**) (i.e., *E_V_*_,cal_: 1440 J/mL).

**Figure 12:**
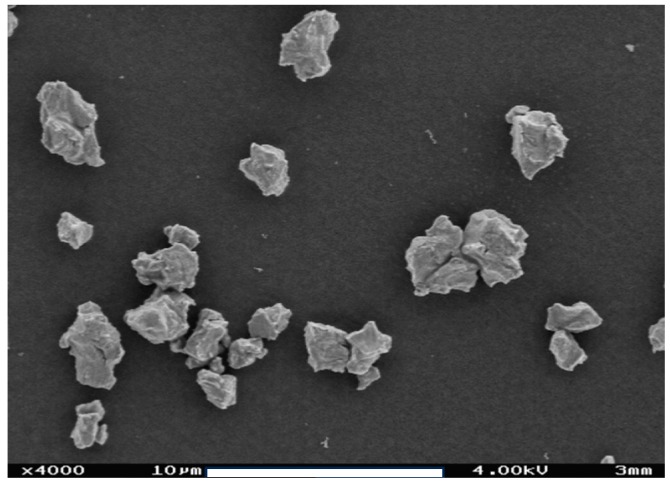
Scanning electron microscope image of wear particles from the sonotrode tip. Abrasion particles were collected from the sediment of a silica suspension sample after high USD energy (i.e., *E_V_*_,cal_: 1440 J/mL).

Figure 11a,b show the effect of high dispersion energy on the sonotrode’s tip if delivered over prolonged period. Wear particles ablated from the tip contaminate suspension and can observed as a black sediment (i.e., coarse titanium particles) and/or as a well as a grey discoloration of the suspension (Figure 11c). Figure 12 shows a SEM picture of sonotrode abrasion particles collected from the bottom of silica suspension. As shown in a previous study, the abrasion of the ultrasonic probe and sample contamination occurs in the moment of ultrasonication; the number of particles increases linearly with time [20]. Furthermore, it was shown that in suspension of pyrogenic silica (PS, 1 wt.-%) at a dispersion energy >171 J/mL sonotrode wear particles contribute to the PSD and interfere with the sample analysis by LD [20]. Nevertheless, this widespread sonicator type is superior to other indirect sonicator types (e.g., ultrasonic bath, cup horn) [36,69] due to its high effectiveness of USD and with regard to the best possible disintegration of agglomerates and aggregates in a short time [20]. Therefore, a restriction of the USD energy appears reasonable. To remove larger particles at lower dispersion energy, e.g., from PS suspensions, we developed a dispersion protocol combining stirring, USD and filtration steps (see Section 3.3).

### 3.3. Sample Preparation with Size-Selective Filtration

**Figure 13:**
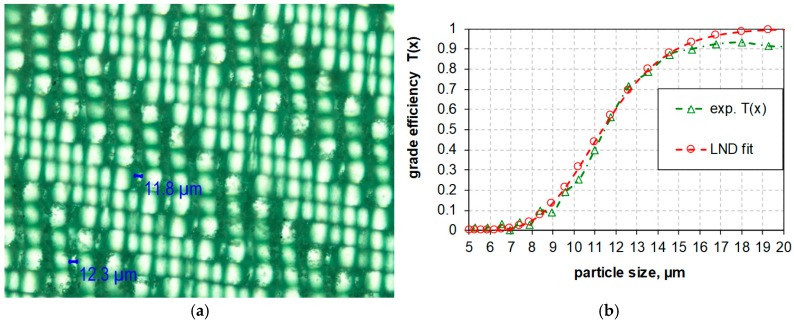
(**a**) Light microscope image of polymer gaze for size-selective suspension filtration and (**b**) *T*(*x*): grade efficiency function after gaze test filtration with glass spheres.

Testing the in vitro toxicity of nanomaterials requires that the size distribution of particles in cell culture media is well defined. With respect to inhalation exposure, which may be tested by the alveolar macrophage assay [24], larger non-respirable coarse particles need to be removed so that a mass-per-volume- or surface-per-volume dose metrics can be applied [15]. Ideally, particle size distribution should reflect inhalable fractions with aerodynamic diameters smaller than 4 μm. However, as outlined above for paddle stirring (PS), this would require high ultrasonic energy and bears the risk of metal particle contamination (see Figure 11 and Figure 12). To circumvent this risk, a size classification by controlled filtration (with 100% fines penetration) was developed, using a commercially available nylon gaze with a pore size of nominally 5 μm (Bückmann, Germany). Figure 13 shows the grade efficiency function *T*(*x*) demonstrating that glass spheres below 7 μm can freely permeate the filter, whereas spheres larger than 15 μm were retained.

The theoretical volume weighted cumulative distribution function of the filtrate *Q*_3,f_(*x*) was derived from the experimentally determined grade efficiency function *T*(*x*) and the feed distribution *Q*_3,*i*_(*x*):

(4) $$\Delta Q_{3,\;f}(x)=(1-T(x))\cdot \left(\frac{\Delta Q_{3,i}(x)}{1-\sum T(x)\cdot \Delta Q_{3,i}(x)}\right)$$

**Figure 14:**
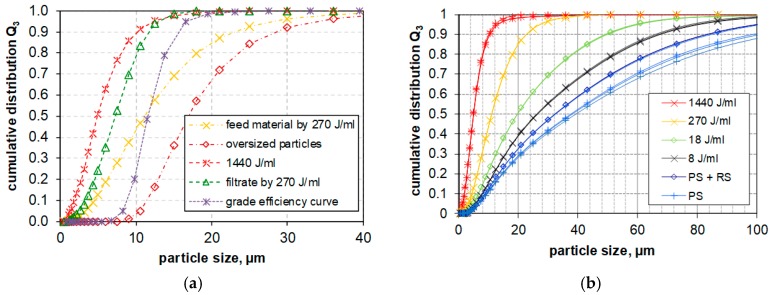
(**a**) filter grade efficiency curve and calculated results for penetrated (filtrate) and retained particle size distributions from feed size distribution (yellow) in comparison to not filtered but with 1440 J/mL dispersed sample (**b**) Evolution of PSD during ultrasonication of precipitated silica (440 m^2^/g); cumulative distribution functions measured by LD.

In the next step ultrasonication and filtration where combined to prepare a suspension suitable for in vitro testing. The method is shown exemplarily for the PS used in this study: the powder was suspended (1 mg/mL) in de-ionized water by means of a magnet stirrer (700 rpm, 10 min). Thereafter the PS sample was filtrated by gaze filter with a grade efficiency curve shown in Figure 13b and a cut-off size of 11.5 μm. The filtrate of the silica sample was then dispersed with *E_V_*_,cal_: 18 J/mL and *E_V_*_,cal_: 270 J/mL. Results were expressed in Figure 14a as cumulative particle volume curves (Q3, green curve) and compared to the effects of progressive dispersion energy on the size distribution of non-filtered PS (red curve). 

Figure 14a shows the advantage of combined moderate ultrasonication by 270 J/mL with filtration (green line) to remove particles larger than 11 μm in comparison with the high ultrasonic energy result (red line), including possible sample pollution. LD results (Figure 14b) show that low energies (i.e., weak dispersion (PS), moderate dispersion (RS) without ultrasonication) leave a considerable amount of micrometer-sized agglomerates in the suspension, whereas ultrasonic dispersion with 8, 18, 270 and 1440 J/mL progressively reduced micrometer-sized agglomerates.

## 4. Conclusions

The effect of dispersion energy on particle size distribution of nanomaterial suspensions depends not only on a defined dispersion procedure (e.g., dispersion time, sample volume) but also on the silica types (e.g., morphology). 

Ultrasonic dispersion energy density is a main parameter for comparability of sample preparation protocols. Sonication is limited by sample pollution with wear particles from the probe. Therefore, upper limit dispersion energy density values must be determined. In the case of silica it is recommended to apply dispersion energy density only up to 300 J/mL.

The resulting particle size distributions strongly depend on the type of silica. Fumed SAS reach PSDs in the submicron range even at low values of ultrasonic energy density; continued sonication leads to a steady, yet slight size reduction. Gel and colloidal SAS are hardly or even adversely affected by increasing ultrasonic dispersion energies. The PSDs of precipitated SAS strongly depends on the increasing ultrasonic dispersion energy, changing constantly to smaller sizes.

Additional size-selective filtration can remove the large and settling particles without the risk of sample contamination by too high ultrasonic energy dispersion. A combination with the above-described ultrasonic dispersion provides a general SOP for the preparation of well-defined suspensions of SAS nanoparticles for in vitro toxicological tests.

## Figures and Tables

**Table 1:** Published protocols of ultrasonic dispersing (USD) specifically designed for application to nanostructured materials; characteristic parameters including the (range of) inserted energy density.

Protocol	Sample Volume	Dispersing Time	(Calorimetric) Energy Density
Tantra 2016 [42]; Pradhan 2016 [43]	6 mL	16 min	1176 J/mL
Rasmussen et al., 2013 [35]	15 mL 10 mL	10 min 16 min	500–400 J/mL 2500 J/mL
Taurozzi et al., 2012 [44]	50 mL	5 min	300 J/mL
Jensen et al., 2011 [33]	6 mL	16 min	3140 J/mL
Bihari et al., 2008 [45]	1 mL	1 min	420 J/mL
Mandzy et al., 2005 [46]	-	Time frames (2 h)	5700 J/mL
Pohl et al., 2005 [37]	10–42 mL	17–630 s	400–30,000 J/mL
Pohl et al., 2004 [47]	3–6 mL	-	100–2000 J/mL

**Table 2:** SAS properties.

SAS Type Internal Code	Fumed Silica F-3	Precipitated Silica P-2	Silica Gel G-1	Colloidal Silica C-1
BET ^1^ (m^2^/g)	300	440	700	200 ^2^
solid content for suspensions (wt.-%)	-	-	-	40
pH ^3^	5	6.5	4.4	9.7
electric conductivity (μS/cm) at 25 °C	4	160	55	4771.6

^1^ BET: Surface measured according to Brunauer, Emmet and Teller [51,52]. ^2^ measured from freeze dried material. ^3^ suspended in ultra-pure water (1 wt.-%, 25 °C).

**Table 3:** Technical data of ultrasonic dispersion instruments operated at approximately 20 kHz.

Model	Vibra-Cell 72412 ^1^	UDS751 ^2^	SONIFIER 450D ^3^
Code	V	T	B
company	Sonics and Materials	Topas GmbH	Branson Ultrasonics
normal capacity (W)	600	200	400
tip diameter (Ø, mm)	13 19	3 7 14	5 13
amplitude (%)	0–100	0–100	10–100

^1^ Vibra-Cell 72412 (Sonics & Materials, Newtown, CT, United States). ^2^ UDS751 (Topas GmbH, Dresden, SN, Germany). ^3^ SONIFIER 450D (Branson Ultrasonic Corporation, Danbury, CT, United States).
